# miRNA profiling identifies deregulated miRNAs associated with osteosarcoma development and time to metastasis in two large cohorts

**DOI:** 10.1002/1878-0261.12154

**Published:** 2017-12-01

**Authors:** Gitte B. Andersen, Alice Knudsen, Henrik Hager, Lise L. Hansen, Jörg Tost

**Affiliations:** ^1^ Department of Biomedicine Aarhus University Denmark; ^2^ Laboratory for Epigenetics and Environment Centre National de la Recherche en Génomique Humaine CEA ‐ Institut de Biologie Francois Jaçob Evry France; ^3^ Department of Pathology Aarhus University Hospital Denmark; ^4^ Department of Pathology Vejle Hospital Denmark

**Keywords:** epigenetics, metastasis, miRNA, osteosarcoma

## Abstract

Osteosarcoma (OS) is an aggressive bone tumor primarily affecting children and adolescents. The etiology of OS is not fully understood. Thus, there is a great need to obtain a better understanding of OS development and progression. Alterations in miRNA expression contribute to the required molecular alterations for neoplastic initiation and progression. This study is the first to investigate miRNA expression in OS in a large discovery and validation cohort comprising a total of 101 OS samples. We established the signature of altered miRNA expression in OS by profiling the expression level of 752 miRNAs in 23 OS samples using sensitive LNA‐enhanced qPCR assays. The identified miRNA expression changes were correlated with gene expression in the same samples. Furthermore, miRNA expression changes were validated in a second independent cohort consisting of 78 OS samples. Analysis of 752 miRNAs in the discovery cohort led to the identification of 33 deregulated miRNAs in OS. Twenty‐nine miRNAs were validated with statistical significance in the second cohort comprising 78 OS samples. miRNA/mRNA targets were determined, and 361 genes with an inverse expression of the target miRNA were identified. Both the miRNAs and the identified target genes were associated with multiple pathways related to cancer as well as bone cell biology, thereby correlating the deregulated miRNAs with OS tumorigenesis. An analysis of the prognostic value of the 29 miRNAs identified miR‐221/miR‐222 to be significantly associated with time to metastasis in both cohorts. This study contributes to a more profound understanding of OS tumorigenesis, by substantiating the importance of miRNA deregulation. We have identified and validated 29 deregulated miRNAs in the – to our knowledge – largest discovery and validation cohorts used so far for miRNA analyses in OS. Two of the miRNAs showed a promising potential as prognostic biomarkers for the aggressiveness of OS.

**Abbreviations**
FF: fresh frozen
FFPE: formalin‐fixed paraffin‐embedded
HE: hematoxylin/eosin
IPA: Ingenuity Pathway Analysis
KEGG: Kyoto Encyclopedia of Genes and Genomes
miRNA: microRNA
MSC: mesenchymal stem cell
OB: osteoblast
OS: osteosarcoma
PCA: principal component analysis
QC: quality control
UTR: untranslated region

## 1 Introduction

Osteosarcoma (OS) is an aggressive tumor of the bone most frequently affecting children and adolescents. OS is often diagnosed at a very late stage, as the first indication of the malignancy is pain, which is often mistaken for more common conditions such as ‘growing pains’ (McCarville, 2009). The late identification of OS leads to 15–25% of the patients exhibiting distant metastases at the time of diagnosis (Bielack *et al*., 2002). The five‐year survival rate of OS patients with no detectable metastases at the time of diagnosis is 65–70% (Bielack *et al*., 2002), whereas patients with relapsing disease or detectable metastases at the time of diagnosis have only a five‐year survival rate of 10–40% (Kager *et al*., 2003; Meyers *et al*., 1993).

OS is characterized by diverse cytogenetic alterations and exhibits changes in multiple different pathways with substantial cell‐to‐cell variation (Bridge *et al*., 1997). The etiology of OS is not well understood and the only prognostic markers are the absence of metastases at the time of diagnosis and the response to neoadjuvant chemotherapy measured as the extent of tumor necrosis (Wang, 2005). However, the characteristic onset of high‐grade neoplasms in young people indicates a consistent, but yet undetected, alteration or group of alterations defining OS.

Cancer initiation and progression is controlled by both genetic and epigenetic events (Hanahan and Weinberg, 2011). Epigenetic changes are particularly important for childhood cancers, which are often characterized by a low mutational load. Molecular subtypes, clinical heterogeneity, and in some cases the prediction of the disease course have been associated with epigenetic changes. This has been demonstrated for ependymomas (Mack *et al*., 2014) and more recently Ewing sarcoma (Sheffield *et al*., 2017), which are characterized by no or the same recurrent genetic aberration, respectively. The genetic components of OS have been intensively studied (Bishop *et al*., 2016; Kansara *et al*., 2014); however, the elucidation of the epigenetic changes is still in its early stage. Gene expression is regulated by numerous mechanisms and molecules, including microRNAs (miRNAs). These are small noncoding RNA molecules (22–25 nucleotides), regulating gene expression post‐transcriptionally by blocking mRNA translation and/or altering the stability of the mRNA by binding perfectly or with mismatches to the 3′‐untranslated region (UTR) of the mRNA (Stark *et al*., 2003). Each miRNA can potentially directly or indirectly regulate more than 100 target genes and thereby affect the expression of both tumor suppressors and oncogenes (Brennecke *et al*., 2005). Furthermore, miRNAs function as classical tumor suppressors and oncogenes/oncomiRs as specific miRNA alterations are associated with carcinogenesis. Deregulation of miRNA expression has been identified in numerous cancers emphasizing the importance of miRNAs in carcinogenesis and their potential as diagnostic, prognostic, and therapeutic means (Calin and Croce, 2006; Negrini *et al*., 2009).

miRNAs are also important regulators of osteogenic signaling pathways and are implicated in normal osteoblast (OB) growth and differentiation (Inose *et al*., 2009; Lian *et al*., 2012). Hence, changes in this balance will affect proliferation of bone cells and may contribute to OS development. The impact of alterations in miRNA expression patterns in OS development has been addressed in several studies (Duan *et al*., 2011; Jones *et al*., 2012; Lulla *et al*., 2011; Maire *et al*., 2011; Namlos *et al*., 2012; Thayanithy *et al*., 2012; Won *et al*., 2013; Zhang *et al*., 2015). However, none of the results are consistent across the studies, which may reflect different methodologies and normalization strategies, as well as low sample numbers. Most studies have evaluated a single miRNA, but a few studies have established a miRNA expression profile of OS by profiling several hundred miRNAs (Duan *et al*., 2011; Jones *et al*., 2012; Lulla *et al*., 2011; Maire *et al*., 2011; Namlos *et al*., 2012; Won *et al*., 2013; Zhang *et al*., 2015). However, all studies have analyzed a limited number of OS samples ranging from 2 to 18.

In this study, we established the signature of miRNA expression alterations in OS by profiling the expression of 752 miRNAs in 23 OS samples using a highly sensitive qPCR technology. Significantly deregulated miRNAs were validated in an independent cohort comprising 78 OS samples. Both the miRNAs and identified target mRNAs were evaluated for their relation to cancer development and progression, as well as bone cell biology.

## 2 Materials and methods

### 2.1 OS samples

Fresh‐frozen diagnostic biopsies of primary tumors from 23 patients diagnosed with OS were collected from the archives of the Institute of Pathology, Aarhus University Hospital (Table 1). The biopsies were immediately snap‐frozen and stored at −80 °C. 25–50 tissue sections of 10 μm were used for RNA extraction for each sample. Tissue sections were hematoxylin/eosin (HE)‐stained for every fifth 10‐μm tissue section cut to confirm the presence of tumor cells.

**Table 1:** Clinical and pathological data for 23 fresh‐frozen and 78 FFPE primary OS samples analyzed

Patient characteristics	Number of patients (%)	
	Discovery cohort	Validation cohort
Total	23 (100)	78 (100)
Gender		
Male	11 (48)	43 (55)
Female	12 (52)	35 (45)
Age (years)		
Median age	17	25.0
Mean age	27.9	32.0
Range	9–76	5–82
Primary site		
Femur	8 (35)	27 (35)
Tibia	10 (44)	22 (28)
Fibula	1 (4)	2 (2)
Others	4 (17)	27 (35)
Metastasis at diagnosis		
Yes	8 (35)	33 (42)
No	15 (65)	45 (58)
Histology		
Osteoblastic	9 (39)	30 (39)
Chondroblastic	9 (39)	22 (28)
Telangiectasic	3 (13)	4 (5)
Other^a^	2 (9)	22 (28)
Chemotherapy treatment		
< 40 years: Cisp., doxo., meth.	15 (65)	44 (56)
> 40 years: Cisp., doxo.	2 (9)	7 (9)
Unknown	6 (26)	27 (35)
Tumor necrosis after chemotherapy		
Good responders (> 90% necrosis)^b^	9 (39)	17 (22)
Poor responders (< 90% necrosis)^c^	8 (35)	34 (43)
Unknown	6 (26)	27 (35)
Survival (months)		
Mean survival	75.5	81.7
Range	1–175	1–282
Long‐term survival		
< 5 years	7 (30)	30 (39)
> 5 years	12 (52)	42 (54)
Not evaluated^d^	4 (18)	6 (7)
Type of sample analyzed		
Biopsy	23 (100)	66 (85)
Larger tumor specimen	0 (0)	12 (15)

Cisp., cisplatin; doxo., doxorubicin; meth., methotrexate.

a All OS samples were high‐grade tumors except for two of the FFPE samples, which were parosteal OS.

b Of the good responders, one patient in the discovery cohort and one in the validation cohort were > 40 years.

c Of the poor responders, one patient in the discovery cohort and six in the validation cohort were > 40 years.

d Patients with < 5 years since diagnosis or who died of other causes than OS.

**Figure 1:**
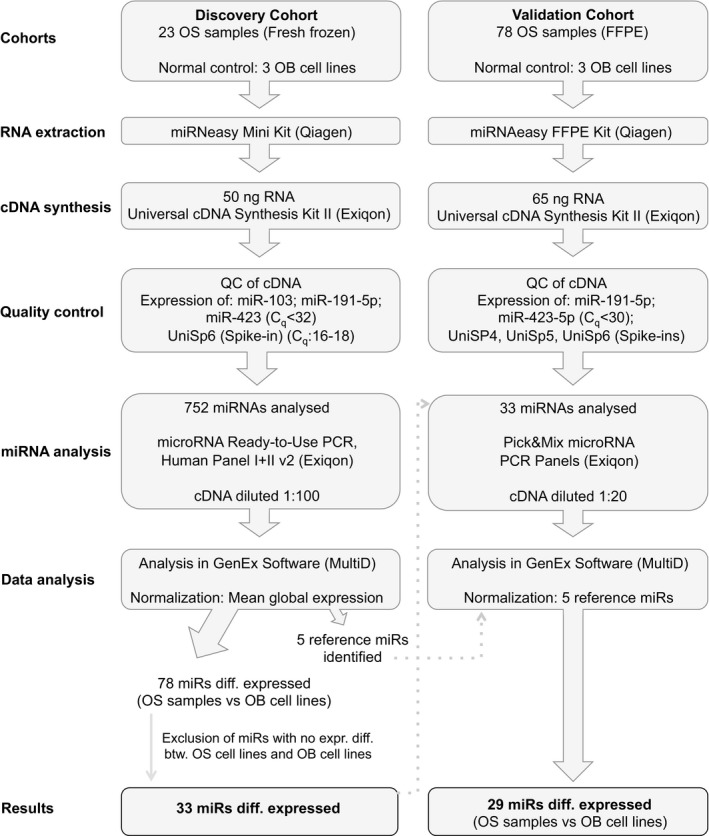
A detailed outline of the different processes and analyses performed for the OS discovery and validation cohort. QC, quality control; diff., differential; btw., between.

Formalin‐fixed, paraffin‐embedded (FFPE) samples from 78 primary tumors from patients diagnosed with OS were collected from the archives of the Institute of Pathology, Aarhus University Hospital (Table 1). A complete outline of the different procedures and analyses performed with each cohort is shown in Fig. 1.

The Local Ethical Committee, The Central Denmark Region, approved this study (journal number 1‐10‐72‐521‐12).

### 2.2 Cell lines

Five OS and three OB cell lines were used in this study. The OS cell lines CRL‐1543, CRL‐1427, CRL‐2098, HTB‐85, and HTB‐96 and the OB cell line CRL‐11372 were purchased from American Type Culture Collection (ATCC, Manassas, VA, USA). The OB cell line C‐12720 was purchased from PromoCell (Heidelberg, Germany), and the OB cell line HO‐f‐4610 was purchased from SanBio (Uden, The Netherlands). HTB‐96 and HTB‐85 were cultured in McCoy's 5a medium modified. CRL‐1427 and CRL‐1543 were cultured in Eagle's minimum essential medium. CRL‐2098 was cultured in RPMI‐1640. All OS cell lines were supplemented with 40 mg·mL^−1^ streptomycin, 240 mg·mL^−1^ penicillin (400 000 U·mL^−1^), and 10% fetal bovine serum, except for HTB‐85, which was supplemented with 15% fetal bovine serum. CRL‐11372 was cultured in 1 : 1 mixture of Ham's F12 medium and Dulbecco's modified Eagle's medium without phenol red, supplemented with 0.3 mg·mL^−1^ G‐418 and 10% fetal bovine serum. C‐12720 was cultured in OB growth medium + supplement mix (PromoCell). HO‐f was cultured in OB medium (ScienCell Research Laboratories, Carlsbad, CA, USA), and the flasks were coated with poly‐L‐lysine solution bioreagent 0.01% (Sigma Aldrich, St Louis, MO, USA).

All eight cell lines were cultured in a humidified atmosphere of 5% CO_2_ at 37 °C, except for CRL‐11372, which was cultured at 34 °C.

### 2.3 Total RNA extraction from fresh‐frozen OS samples and cell lines

Total RNA including miRNAs was extracted from the 23 fresh‐frozen OS samples and the eight cell lines. The tissue was homogenized using a TissueLyser bead mill and one tungsten carbide bead (3 mm) per sample. RNA extraction was performed using the miRNeasy Mini Kit (Qiagen, Hilden, Germany) according to the manufacturer's instructions until each sample was separated into three phases. The upper aqueous phase was transferred to a new tube and total RNA was purified using a QIAcube automated device (Qiagen) according to the manufacturer's instructions including an on‐column DNase digest. RNA was quantified using a NanoDrop ND‐2000c spectrophotometer (Thermo Fisher Scientific, Waltham, MA, USA), and the quality was assessed using the RNA 6000 Nano Kit (Agilent Technologies Inc., Santa Clara, CA, USA) on an Agilent 2100 BioAnalyzer.

### 2.4 RNA extraction from FFPE samples

Four tissue sections of 10 μm from each FFPE sample were used for RNA extraction. RNA was extracted using the miRNeasy FFPE Kit (Qiagen) according to the manufacturer's instructions (Fig. 1). One microlitre Spike‐in (UniSp2, UniSp4, and UniSp5), from the RNA Spike‐in kit, UniRT (Exiqon, Vedbæk, Denmark), was added to the PKD buffer during the purification.

### 2.5 cDNA synthesis and miRNA expression analysis of fresh‐frozen OS samples

For the initial miRNA profiling analysis, 50 ng of total RNA from 23 fresh‐frozen OS samples, two OS, and three OB cell lines was reverse‐transcribed using the miRCURY LNA™Universal RT microRNA PCR kit (Exiqon) in a Mastercycler^®^ pro S (Eppendorf, Hamburg, Germany) in a 50 μL reaction volume, comprising the Universal cDNA synthesis kit II, RNA Spike‐in kit (Exiqon) (Fig. 1). Quality control (QC) was performed according to the manufacturer's instructions using the ExiLENT SYBR^®^ Green master mix kit (Exiqon), using 1 : 80 diluted cDNA. Quality of the cDNA synthesis and PCR amplification were evaluated through the detection of miR‐103, miR‐191‐5p, miR‐423, and UniSp6 at stable *C*_q_ levels (*C*_q_ < 32) (Fig. 1).

The miRNA profile was established using 45 ng cDNA from each sample by qPCR using the microRNA Ready‐to‐Use PCR, Human panel I and panel II v2 analyzing 752 miRNAs (Exiqon) on a Lightcycler^®^ 480 V2 Real‐Time PCR System (Roche Applied Science, Penzberg, Germany) (Fig. 1). The plates were set up using a Bravo Automated Liquid Handling Platform (Agilent Technologies Inc.).

### 2.6 Validation of miRNA expression in OS FFPE samples

A total of 65 ng RNA (5 ng·μL^−1^) from the OS FFPE samples and the cell lines was reverse‐transcribed in 20 μL reactions using the Universal cDNA Synthesis kit II (Exiqon) and a 2720 Thermal Cycler (Thermo Fisher Scientific) (Fig. 1). Reverse transcription was performed in duplicate for each sample. All the subsequent amplifications were performed using a Lightcycler^®^ 480 Real‐Time PCR System (Roche Applied Science). QC of the cDNA synthesis and PCR amplification were performed according to the manufacturer's instructions (Exiqon), with slight modifications; the cDNA was diluted 1 : 20 to ensure the detection of lowly expressed miRNAs. Spike‐ins were included in the RNA purification and RT step using the RNA Spike‐in kit, UniRT (Exiqon). hsa‐miR‐191‐5p, hsa‐miR‐423‐5p, UniSp4, UniSp5, UniSp6, and Cel‐miR‐39‐3p were used for QC of the RNA purification and cDNA synthesis (Fig. 1). UniSp4 and UniSp5 RNA spike‐in controls were used for QC of RNA purifications. All primary OS FFPE samples, except one, had *C*_q_ values < 37 for UniSp5. UniSp6 and Cel‐miR‐39‐3p spike‐in control primers were used for QC of the cDNA synthesis and to rule out PCR inhibition, as a higher concentration of cDNA was used in the analysis, than described in the manufacturer's instructions (65 ng RNA instead of 20 ng for the cDNA synthesis and 1 : 20 dilution of the cDNA instead of 1 : 100). These conditions were optimized for these amounts to ensure adequate amplification from the FFPE samples. All primary OS FFPE samples had *C*_q_ values between 16.5 and 18 for UniSp6. Negative controls included in the cDNA synthesis were a no RNA template and a no RT enzyme.

miRNA expression analysis was performed for 78 FFPE primary OS samples, five OS, and the three OB cell lines using 5 μL 1 : 20 diluted cDNA from each sample using a custom Pick&Mix microRNA PCR Panel, 384 well Ready‐to‐Use (Exiqon) (Fig. 1). Five reference miRNAs, hsa‐miR‐148b‐3p, hsa‐miR‐185‐5p, hsa‐miR‐191‐5p, hsa‐miR‐423‐5p, and hsa‐miR‐425‐3p, were selected based on the results from the discovery cohort, using both geNorm (Vandesompele *et al*., 2002) and NormFinder (Andersen *et al*., 2004). All 78 OS FFPE samples had *C*_q_ values < 30 for the reference miRNAs.

### 2.7 Gene expression analysis

Genomic RNA expression analysis was performed using the SurePrint G3 Human 8X60K One color Microarrays (Agilent), which analyzes 27958 Entrez Gene RNAs and 7419 lincRNAs. Hundred nanogram RNA from each sample of the 23 fresh‐frozen OS primary tumors, five OS cell lines, and three OB cell lines was analyzed as per the manufacturer's instructions. The Agilent G3 Microarrays were scanned using the NimbleGen MS200 scanner with Multi‐TIFF as File Settings Control, a single scan area was selected, and the entire feature area was captured. Two‐micrometre resolution was selected. The red channel was omitted from the analysis. Grid alignment was determined manually. Data were extracted using the Agilent Feature Extraction 11.5.1.1. The raw data were preprocessed using ArrayStar v12.1.0 build 134 (DNASTAR, Madison, WI, USA) and normalized using the quantile normalization method and log_2_‐transformed. Genes were considered differentially expressed when the difference between OS samples and OB cell lines was > 2‐fold and with a Bonferroni‐corrected *P*‐value < 0.05. Only genes with an average normalized probe intensity of log_2_ > 6 in OB cell lines were considered expressed.

### 2.8 Data analysis

miRNA expression analysis was performed using the genex software version 5.4.2.128 (MultiD, Göteborg, Sweden). Raw data (*C*_q_ values) for the 752 miRNAs analyzed using the discovery cohort were imported and merged (Table S1). Each sample has been run with two 384‐well plates containing different miRNAs. Hence, the raw data contain numerous NA values for the miRNAs not present in the respective plates. The mean global expression was used for normalization of the Human Panel I+II v2. The limit for a detectable miRNA was set to *C*_q _< 37. Log_2_‐normalized data are available in Table S2. Pick&Mix microRNA PCR Panels were normalized using five selected stably expressed reference genes (hsa‐miR‐148b‐3p, hsa‐miR‐185‐5p, hsa‐miR‐191‐5p, hsa‐miR‐423‐5p, and hsa‐miR‐425‐3p) (Fig. 1). Raw data (*C*_q_ values) for the 33 miRNAs analyzed in the validation cohort are available in Table S3. The raw data have been imported and merged using the genex software version 5.4.2.128 (MultiD). Log_2_‐normalized data for the validation cohort are available in Table S4.

The statistical software r v.3.0.0 (R Core Team 2013) was used for unsupervised hierarchical clustering using the ‘Ward’ method as distance measure with an in‐house‐developed script. Pathway and network analyses were performed using Ingenuity^®^ Pathway Analysis (IPA^®^; Qiagen), the Kyoto Encyclopedia of Genes and Genomes (KEGG) (Kanehisa and Goto, 2000; Kanehisa *et al*., 2014), and DIANA miRPath.v3 (Vlachos *et al*., 2015). For the IPA core analysis, the KEGG pathway analysis, and the DIANA pathway analysis, human was selected as the species analyzed.

Validated miRNA gene targets were established using miRWalk2.0 (Dweep and Gretz, 2015) with the identification of miRNA binding sites in the 3′UTR. The generated gene lists of validated miRNA gene targets were compared with a list of genes comprising > 2‐fold change (*P*‐value < 0.05) in expression between 23 OS samples and three OB cell lines using Venn analysis (G. B. Andersen, T. E. Kjeldsen, F. Busato, C. Dong, J. Q. D. Tran, A. Daunay, D. B. Hussmann, F. Jehan, V. Geoffroy, J. F. Deleuze, H. Hager, E. Willerslev, L. L. Hansen & J. Tost, in preparation). Boxplots, Mann–Whitney *U*‐tests, and Kaplan–Meier analysis were made with the graphpad prism 7 Software (GraphPad Software Inc., La Jolla, CA, USA). For Kaplan–Meier analysis, the log‐rank (Mantel–Cox) test was used for the determination of *P*‐values. For the prognostic analyses (survival and time to metastasis), median expression was used to subdivide the samples into high and low expression groups.

### 2.9 Data accessibility

All miRNA data generated and analyzed during this study are included in this published article (Tables S1–S4 including both raw and log_2_‐normalized data). The normalized log_2_ expression data for the 361 genes analyzed in this study are provided in Table S5. The raw gene expression dataset used in the current study is part of another ongoing study and will be published and deposited in a public repository separately. Meanwhile, these data are available from the corresponding author upon request.

## 3 Results

In the present study (Fig. 1), we investigated changes in miRNA expression of 752 miRNAs in primary OS and OS cell lines compared to the miRNA expression levels of OB cell lines. OB cell lines were used as a reference as OBs/osteoprogenitor cells are considered to represent the cell of origin for OS (Abarrategi *et al*., 2016). The 33 miRNAs identified as differentially expressed in the OS discovery cohort were validated in an independent validation cohort comprising 78 primary OS samples.

### 3.1 Overall miRNA expression

The relative expression levels of 752 miRNAs were evaluated in 23 primary OS samples, two OS, and three OB cell lines. Following normalization, using the global mean, a total of 339 and 202 miRNAs were reliably detected in more than 40% and 90% of the samples, respectively. The frequency of missing data was calculated based on all samples (both OS samples and OB cell lines). Further analyses were performed using the dataset with the expression of 339 miRNAs to ensure analysis of all miRNAs important for OS etiology. If using a 90% threshold, miRNAs not expressed in OB cell lines or miRNAs downregulated/depleted in OS samples could be removed, thereby excluding biologically relevant miRNAs.

**Figure 2:**
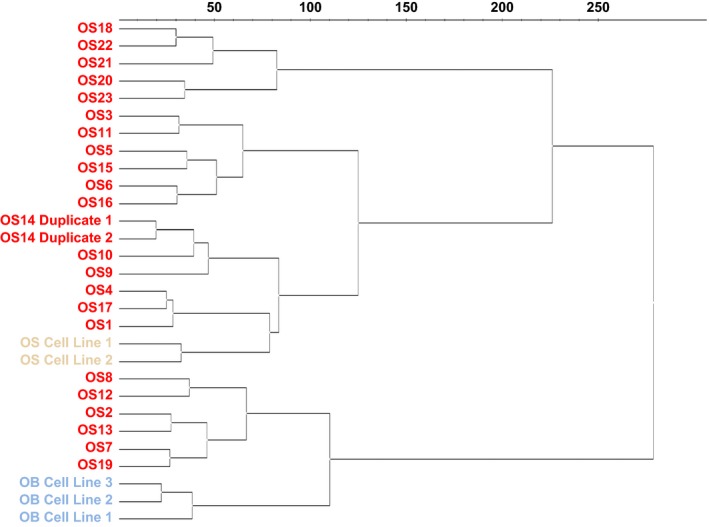
Unsupervised hierarchical cluster analysis. The expression value (log_2_) for each detectable miRNA (*C*_q _< 37) was used to determine the clustering of the samples. Red: primary OS samples; yellow: OS cell lines; blue: OB cell lines.

An unsupervised hierarchical clustering was performed using the log_2_‐transformed expression values for the 339 miRNAs (Fig. 2). The samples separated into two main clusters, of which one contained 17 OS samples and the two OS cell lines. Even though the OS cell lines were in a cluster with OS samples, the cell lines kept the closest mutual resemblance, indicating a cell line similarity stronger than the cell origin. The other cluster contained six OS samples and the three OB cell lines. There was no difference between tumor content or tumor/patient characteristics in these six OS samples compared with the rest of the OS cohort.

The two observed clusters could not be explained with the available clinical and histopathological data as no separation in terms of the primary tumor site, histology, age, gender, or metastatic status was observed, even though a smaller group containing five OS samples separated from the other OS samples (Fig. S1). These samples had a slightly higher miRNA detection level, which might explain the separation. Of note, these samples were different from the six OS samples grouping apart in the cluster.

### 3.2 Identification of differentially expressed miRNAs in OS

**Figure 3:**
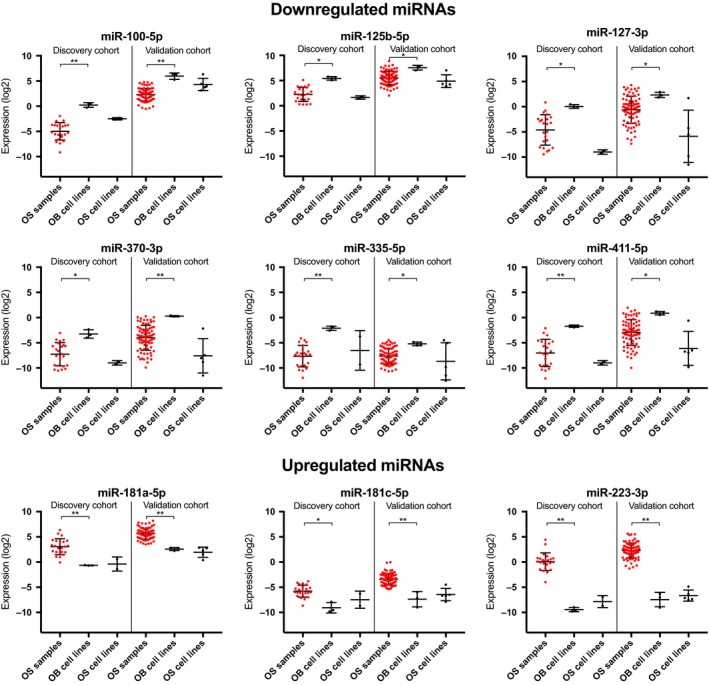
Boxplots of the expression of nine differentially expressed miRNAs. Red: OS samples. *P < 0.05; **P < 0.001.

When comparing expression profiles between OS samples and OB cell lines, 76 miRNAs were identified as differentially expressed (*P*‐value < 0.05) (Table S6). As OB cell lines were used as the normal control, miRNAs with no expression change between OS and OB cell lines were excluded to avoid expression changes caused by cell culture and not as a result of cell origin. This provided a list of 33 miRNAs with deregulated expression levels, of which 26 were significantly downregulated and seven were upregulated in the OS samples compared to OB cell lines (Table 2, Fig. 3 and Fig. S2A,B). Four of these miRNAs (miR‐181a‐5p, miR‐181b‐5p, miR‐30e‐3p, and miR‐92b‐3p) did not exhibit an expression difference between OS and OB cell lines, but were, nonetheless, selected for further validation as miR‐181a‐5p, miR‐181b‐5p, and miR‐92b‐3p have previously been associated with OS development and miR‐30e‐3p has been shown to be deregulated in several other cancers (Duan *et al*., 2011; Jones *et al*., 2012; Ning *et al*., 2016; Zhu *et al*., 2014). Of the 33 differentially expressed miRNAs, 12 miRNAs have not previously been described as differentially expressed in OS (Table S7). Of the previously described miRNAs, 16 were identified with the same direction of the expression change as in this study, while five miRNAs showed an opposite expression change (Table S7). On the other hand, previous studies using microarrays or sequencing to investigate miRNA expression changes in OS identified 14 miRNAs, which were not part of the 33 validation miRNAs found in this study (each miRNA was identified in three or more studies) (Table S8). However, 10 of the miRNAs were identified as both up‐ and downregulated in different profiling studies. A further investigation of these 14 miRNAs in the OS discovery cohort analyzed in this study showed that six of the miRNAs did exhibit a differential expression in OS samples when compared to OB cell lines, but no difference was observed between OS and OB cell lines; four of the miRNAs did not exhibit a significant difference between OS samples and OB cell lines, and four miRNAs were not present in our normalized data set.

**Table 2:** Expression level of 33 significantly differentially expressed miRNAs in OS samples compared to OB cell lines

miRNA	Discovery cohort			Validation cohort		
	Fold change^a^	*P*‐value	No. OS sign. changed (%)	Fold change^a^	*P*‐value	No. OS sign. changed (%)
Downregulated miRNAs						
**miR‐100‐5p**	−34.75	8.0 × 10^−4^	23 (100)	−12.80	2.34 × 10^−5^	76 (97)
**miR‐221‐3p**	−14.21	8.0 × 10^−4^	23 (100)	−10.79	2.34 × 10^−5^	78 (100)
**miR‐29b‐1‐5p**	−16.45	8.0 × 10^−4^	23 (100)	−17.44	2.34 × 10^−5^	78 (100)
**miR‐125b‐1‐3p**	−13.50	8.0 × 10^−4^	23 (100)	−5.31	9.38 × 10^−5^	76 (97)
**miR‐29a‐5p**	−33.65	8.0 × 10^−4^	23 (100)	−3.60	9.38 × 10^−5^	72 (92)
**miR‐370‐3p**	−14.89	0.0031	20 (87)	−19.62	9.38 × 10^−5^	77 (99)
**miR‐299‐5p**	−19.42	0.0015	22 (96)	−24.42	2.0 × 10^−4^	76 (97)
**miR‐493‐5p**	−25.62	0.0085	20 (87)	−32.46	3.0 × 10^−4^	75 (96)
**miR‐409‐3p**	−31.36	0.0015	22 (96)	−19.60	4.0 × 10^−4^	71 (91)
**miR‐30e‐3p**	−2.91	0.0015	21 (91)	−2.78	0.0012	67 (86)
**miR‐431‐5p**	−4.92	0.0031	19 (83)	−16.77	0.0012	69 (88)
**miR‐432‐5p**	−16.05	0.0031	22 (96)	−22.79	0.0016	74 (95)
**miR‐410‐3p**	−16.83	8.0 × 10^−4^	21 (91)	−14.66	0.0019	70 (90)
**miR‐411‐5p**	−36.54	8.0 × 10^−4^	22 (96)	−14.47	0.0034	67 (86)
**miR‐376c‐3p**	−23.90	0.0031	22 (96)	−16.57	0.0056	61 (78)
**miR‐125b‐5p**	−8.45	0.0015	22 (96)	−4.06	0.0064	61 (78)
**miR‐335‐5p**	−45.39	8.0 × 10^−4^	23 (100)	−5.32	0.0064	64 (82)
**miR‐376a‐3p**	−34.17	0.0085	21 (91)	−15.79	0.0064	65 (83)
**miR‐382‐5p**	−13.97	0.0054	21 (91)	−10.53	0.0135	62 (79)
**miR‐154‐5p**	−13.62	0.0085	20 (87)	−9.04	0.0244	53 (68)
**miR‐222‐3p**	−5.11	0.0123	19 (83)	−2.49	0.0267	54 (69)
**miR‐92b‐3p**	−3.19	0.0085	21 (91)	−2.01	0.0267	58 (74)
**miR‐433‐5p**	−4.16	0.0054	18 (78)	−4.76	0.029	52 (67)
**miR‐127‐3p**	−21.94	0.0054	22 (96)	−7.72	0.04	60 (77)
miR‐34a‐3p	−3.44	0.0085	17 (74)	−2.99	0.0888	5 (5)
miR‐136‐5p	−13.63	0.0315	21 (91)	−2.72	0.248	35 (45)
Upregulated miRNAs						
**miR‐181a‐5p**	12.04	8.0 × 10^−4^	23 (100)	8.38	2.34 × 10^−5^	78 (100)
**miR‐181c‐5p**	9.29	0.0015	20 (87)	15.24	2.34 × 10^−5^	60 (77)
**miR‐223‐3p**	727.23	8.0 × 10^−4^	23 (100)	859.9	2.34 × 10^−5^	78 (100)
**miR‐342‐3p**	12.84	0.0031	17 (74)	3.44	9.38 × 10^−5^	75 (96)
**miR‐378a‐3p**	4.20	0.0177	19 (83)	5.49	3.0 × 10^−4^	74 (95)
miR‐128‐3p	3.22	0.0015	22 (96)	1.06	0.9328	16 (21)
miR‐181b‐5p	4.19	0.0085	14 (61)	2.72	0.1051	25 (32)

Discovery cohort = 23 fresh‐frozen OS samples; validation cohort = 78 FFPE OS samples; miRNAs in bold text = significantly differentially expressed in both cohorts. No., number.

a  Expression change between primary OS samples and OB cell lines.

### 3.3 Validation of differentially expressed miRNAs

The 33 miRNAs identified in the discovery cohort were validated in a larger, independent cohort consisting of 78 primary OS FFPE samples using a custom‐designed Pick&Mix microRNA PCR Panel.

Of the 26 miRNAs with decreased expression, 24 were confirmed in the validation cohort, and of the seven miRNAs with increased expression, five were confirmed, thereby corroborating 88% of our initial findings (Table 2, Fig. 3 and Fig. S2A,B).

### 3.4 Pathways associated with the deregulated miRNAs and their target genes

To determine the possible impact of the 29 differentially expressed miRNAs on OS development, enriched biological functions were explored using Ingenuity Pathway Analysis (IPA) and DIANA Tools mirPath.v3 exploring KEGG pathways. Several biological functions related to cancer development and progression were enriched for the 29 deregulated miRNAs (Table 3).

**Table 3:** Biological functions associated with the 29 deregulated miRNAs in OS. (A) IPA biological functions^a^ and (B) KEGG pathways^b^ identified by DIANA miRPath.v3

(A)	*P*‐value	No. of miRNAs associated
Diseases and disorders		
Cancer^c^	4.14 × 10^−13^	18
Connective tissue disorders	4.14 × 10^−13^	12
Organismal injury and abnormalities	4.14 × 10^−13^	20
Reproductive system disorders	8.23 × 10^−9^	15
Developmental disorder	4.49 × 10^−8^	6
Molecular and cellular functions		
Cell death and survival^c^	2.84 × 10^−4^	5
Cell‐to‐cell signaling and interaction^c^	0.00342	1
Cellular development^c^	0.00342	6
Cellular growth and proliferation^c^	0.00570	6
Cell cycle^c^	0.0125	1

(B)Pathway	*P*‐value	No. of miRNAs associated
Proteoglycans in cancer^c^	2.93 × 10^−13^	20
Prion diseases	1.52 × 10^−10^	15
ECM/receptor interaction^c^	9.02 × 10^−10^	20
Other types of O‐glycan biosynthesis	6.83 × 10^−8^	13
Hippo signaling pathway^c^	1.49 × 10^−7^	20
Glioma^c^	5.18 × 10^−6^	19
Signaling pathways regulating pluripotency of stem cells^c^	5.18 × 10^−6^	21
Steroid biosynthesis	5.38 × 10^−6^	9
Mucin‐type O‐glycan biosynthesis	9.70 × 10^−6^	14
Transcriptional misregulation in cancer^c^	2.95 × 10^−5^	21

a Diseases and molecular functions associated with the 29 deregulated miRNAs identified by IPA.

b KEGG pathways associated with the 29 deregulated miRNAs identified by DIANA miRPath.v3.

c Association with cancer development and progression.

Alterations in miRNA expression may have an extensive effect on gene expression, as one miRNA can directly or indirectly regulate more than 100 target genes by binding to their 3′UTR. Genes targeted by at least one of the 29 differentially expressed miRNAs were therefore identified using miRWalk2.0 analyzing only experimentally validated target genes. Genome‐wide RNA expression changes in OS were established by analyzing the 23 OS samples from the discovery cohort with RNA expression microarrays containing 27958 Entrez Gene RNAs (Andersen *et al*., in preparation). The identified target genes were correlated with genes identified as differentially expressed in the OS samples compared to OB cell lines (> 2‐fold change and *P*‐value < 0.05). This identified 301 genes with upregulated expression in OS samples, targeted by one or more of the identified downregulated miRNAs, as well as 60 genes with decreased expression in the OS samples targeted by one or more of the miRNAs with increased expression in the OS samples (Table S5).

The possible influence of the deregulation of miRNA expression on OS development, associated with the affected target genes, was investigated using IPA and KEGG pathway analysis. The most significant pathways, as well as biological functions associated with disease and cellular functions for validated target genes, are reported in Table 4. For IPA, six of the top ten biological functions and three of the top five pathways were associated with cancer development and progression. Furthermore, one of the biological functions, for which deregulated genes were enriched, was ‘Skeletal Disorders’ and one of the top canonical pathways was ‘Role of Osteoblasts, Osteoclasts, and Chondrocytes in Rheumatoid Arthritis’. For the KEGG analysis, nine of the ten highest‐ranking pathways were related to cancer development and progression. The 27th pathway for the KEGG analysis was ‘Osteoclast Differentiation’, thereby linking bone cell biology and cancer development and progression for the target genes with altered gene expression.

**Table 4:** Pathways and biological functions associated with genes targeted by deregulated miRNAs

IPA canonical pathways	*P*‐value	
Atherosclerosis signaling	5.60 × 10^−7^	
Role of NANOG in mammalian embryonic stem cell pluripotency^a^	2.53 × 10^−6^	
Role of osteoblasts, osteoclasts, and chondrocytes in rheumatoid arthritis^b^	5.59 × 10^−6^	
Role of tissue factor in cancer^a^	1.41 × 10^−5^	
Natural killer cell signaling^a^	1.53 × 10^−5^	

IPA biological functions	*P*‐value	No. of genes associated
Diseases and disorders		
Connective tissue disorders	2.86 × 10^−13^	77
Inflammatory disease	2.86 × 10^−13^	91
Organismal injury and abnormalities	2.86 × 10^−13^	348
Skeletal and muscular disorders^b^	2.86 × 10^−13^	104
Cancer^a^	4.66 × 10^−11^	343
Molecular and cellular functions		
Cellular growth and proliferation^a^	1.28 × 10^−11^	121
Cellular movement^a^	1.70 × 10^−10^	80
Cellular Development^a^	1.05 × 10^−9^	86
Cell‐to‐cell signaling and interaction^a^	5.07 × 10^−8^	60
Cell death and survival^a^	6.10 × 10^−7^	102

KEGG pathways		No. of genes associated
Metabolic pathways^a^		22
MAPK signaling pathway^a^		17
Pathways in cancer^a^		16
Cytokine/cytokine receptor interaction^a^		14
PI3K‐Akt signaling pathway^a^		14
Ras signaling pathway^a^		14
Proteoglycans in cancer^a^		13
Regulation of actin cytoskeleton^a^		12
Rap1 signaling pathway		12
MicroRNAs in cancer^a^		11

IPA and KEGG analysis of the 301 upregulated and 60 downregulated genes identified to be targeted by the 29 deregulated miRNAs.

a Association with cancer development and progression.

b Association with bone cell biology.

**Figure 4:**
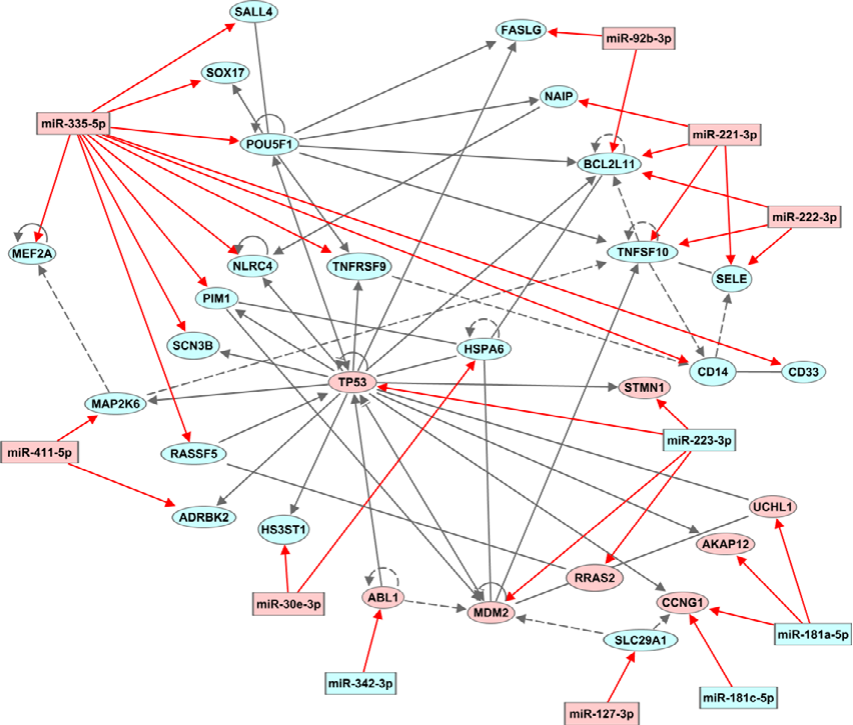
Network of validated target genes. Network of validated target genes associated with the terms ‘Cell Death and Survival, Cancer, Hematological System Development and Function’. The relation of 29 validated target genes (miRWalk2.0) assembled in a network associated with 11 miRNAs altered in OS in this study. Eight genes were downregulated and 21 upregulated. Red: downregulated; blue: upregulated. Gray lines: association between genes; Red lines: association between gene and miRNA; continuous line: direct association; dashed line: indirect association.

An analysis of the biological relations between the target genes identified a network of 21 upregulated and eight downregulated genes significantly associated with ‘Cell Death and Survival, Cancer, Hematological System Development and Function’ (*P*‐value = 10^−42^). The intimate relationship of the 29 genes and the seven downregulated and four upregulated miRNAs validated to target these genes is illustrated in Fig. 4. The identified network comprised five genes (*CCNG1*,* MDM2*,* TNFSF10*,* TP53*, and *UCHL1*) directly associated with the *TP53* pathway. Three additional target genes (*ABL1*,* FASLG*, and *UBD*) not part of the identified network were associated with the *TP53* pathway. Four target genes (*ABL1*,* MDM2*,* STMN1*, and *TP53*) were associated with the *RB* pathway.

### 3.5 miRNAs associated with the metastatic status and response to chemotherapy

To identify miRNAs with an overall difference in expression based on the metastatic status, the discovery cohort was separated into metastatic and nonmetastatic primary OS (8 and 15 OS samples, respectively). This analysis revealed three miRNAs (miR‐29b‐3p, miR‐29c‐3p, and miR‐374a‐5p) significantly differentially expressed between metastatic and nonmetastatic OS samples (Table S9 and Fig. S3). The response to neoadjuvant chemotherapy was analyzed by subdividing the discovery cohort into poor responders (< 90% necrosis after chemotherapy) and good responders (> 90% necrosis after chemotherapy) (8 and 9 OS samples, respectively). This identified miR‐663b as significantly differentially expressed. Also, miR‐664a‐3p showed a strong trend toward differential expression (Table S9 and Fig. S3). However, when these miRNAs were analyzed in the samples from the validation cohort, none of the three miRNAs associated with the metastatic status nor the two miRNAs associated with the chemotherapeutic response displayed a significant difference (Table S9 and Fig. S3).

### 3.6 Prognostic value of the 29 differentially expressed miRNAs

The only prognostic markers for OS are currently absence of metastases at the time of diagnosis and response to chemotherapy. As 25–30% of OS patients develop distant metastases and the five‐year survival rate for patients with relapse is only 10–40%, it is critical to identify new prognostic biomarkers for OS.

We therefore investigated the prognostic value in function of survival and time to development of metastatic disease of the 29 differentially expressed miRNAs. Kaplan–Meier analysis was performed on the discovery cohort divided into two groups based on the median expression (high and low expression groups for each miRNA). For the overall survival analysis, two miRNAs (miR‐128‐3p and miR‐34a‐3p) were borderline significant in the discovery cohort (*P *= 0.0880 and 0.0760, respectively). No significant difference was, however, observed in the larger OS cohort (*P *= 0.5645 and 0.7507, respectively).

**Figure 5:**
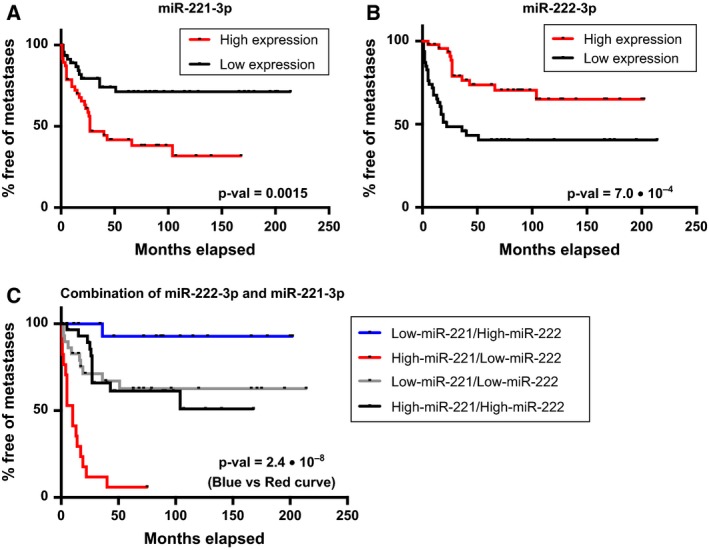
The prognostic value of the expression of miR‐221‐3p and miR‐222‐3p in function of time to metastasis. (A) Kaplan–Meier analysis of time to metastasis when subdividing the OS samples into high/low expression groups for miR‐221‐3p (based on the median expression). (B) Kaplan–Meier analysis of time to metastasis when subdividing the OS samples into high/low expression groups for miR‐222‐3p (based on the median expression). (C) Kaplan–Meier analysis of time to metastasis when combining the expression of both miR‐221‐3p and miR‐222‐3p for each patient. The discovery cohort and validation cohort have been combined for all three analyses. The Kaplan–Meier analysis of the high/low expression groups for the individual analyses of miR‐221‐3p and miR‐222‐3p contained 47 OS samples in each group. For the combined analysis of miR‐221‐3p/miR‐222‐3p expression, the number of OS samples in each group was as follows: 29 OS samples in high‐miR‐221‐3p/high‐miR‐222‐3p; 30 OS samples in low‐miR‐221‐3p/low‐miR‐222‐3p; 17 OS samples in high‐miR‐221‐3p/low‐miR‐222‐3p; and 18 OS samples in low‐miR‐221‐3p/high‐miR‐222‐3p. Data for the individual cohorts are shown in Fig. S4.

Analysis of the miRNA expression in function of time to metastasis, describing the aggressiveness of the OS tumor, identified miR‐222‐3p and miR‐221‐3p as significantly (*P *= 0.0359) and borderline significantly (*P *= 0.0863) associated with time to metastasis in the discovery cohort, respectively (Fig. S4A,C). High expression of miR‐221‐3p and low expression of miR‐222‐3p correlated with a poor prognostic value and earlier development of distant metastases. The association between expression level and poor prognosis in terms of earlier development of distant metastases was highly significant in the validation cohort (*P *= 0.0065 and 0.0074, respectively) (Fig. S4B,D) and when combining results from both the discovery and validation cohorts (*P *= 0.0007 and 0.0015, respectively) (Fig. 5A,B). When combining the expression level of the two miRNAs into one prognostic signature, there was a clear separation between OS patients with low‐miR‐221‐3p/high‐miR‐222‐3p expression and OS patients with high‐miR‐221‐3p/low‐miR‐222‐3p for both the discovery and validation cohorts and in combination (*P *= 2.4 × 10^−8^) (Fig. 5C and Fig. S4E,F).

## 4 Discussion

OS is a complex disease exhibiting alterations in numerous pathways and with major cell‐to‐cell variation. The understanding of the etiology of OS is limited and no common molecular alteration has been identified. This designates the need for a more fundamental understanding of the underlying mechanisms leading to OS development and progression. Alterations in miRNA expression are one of the mechanisms associated with tumor development. As one miRNA potentially regulates the expression of more than 100 target genes, alterations in miRNA expression can have a vast impact on neoplastic initiation and progression. Hence, the focus of this study was the analysis of miRNA expression changes in OS. Profiling of miRNA expression in OS has been addressed in previous studies (Duan *et al*., 2011; Jones *et al*., 2012; Lulla *et al*., 2011; Maire *et al*., 2011; Namlos *et al*., 2012; Thayanithy *et al*., 2012; Won *et al*., 2013; J. Zhang *et al*., 2015). However, the limited number of samples of 2–18 OS samples in the discovery cohorts and 2–12 samples in the respective validation cohorts makes it challenging to draw reliable conclusions from the published data.

We have in this study investigated the expression of 752 miRNAs in 23 OS samples and validated significant findings in 78 additional OS samples. These are, to our knowledge, both the largest discovery and validation cohorts so far used for miRNA expression analysis in OS. The identified miRNA expression signature showed strong statistical significance in both the discovery and the validation cohorts comprising together a total of 101 OS samples. This provides robust evidence of the 29 miRNAs with an altered expression level between OS samples and OB cell lines to be truly deregulated in OS. A difference in miRNA expression between histological subtypes was not identified designating a universal role of the deregulated miRNAs in OS tumorigenesis.

A significant role of miRNAs in OS tumorigenesis was supported by the investigation of biological functions associated with the 29 deregulated miRNAs, revealing a strong correlation with cancer development and progression. This was further substantiated by the investigation of the validated miRNA/mRNA targets, identifying 361 genes with a significantly changed expression level in the OS samples. These genes were associated with multiple pathways and biological functions related to cancer development and progression. Furthermore, both a canonical pathway and biological functions were associated with bone cell pathogenesis linking the cancer‐related pathways with OS tumorigenesis.

A relation between the identified pathways associated with the target genes and the *TP53* pathway, which is often altered in OS, also supported a correlation between the deregulated miRNAs and OS tumorigenesis. The network analysis and target genes comprised a total of eight genes directly associated with the *TP53* pathway (*ABL1*,* CCNG1*,* FASLG*,* MDM2*,* TNFSF10*,* TP53*,* UBD*, and *UCHL1*) and four genes (*ABL1*,* MDM2*,* STMN1*, and *TP53*) associated with the RB pathway. Hence, the altered miRNAs identified in this study fit well with the current view of OS pathogenesis that the *TP53* and RB pathways are the two major pathways involved in OS development and progression. Many potential functional miRNA/mRNA relationships were identified in this study. These need to be further investigated to determine their function and involvement in OS tumorigenesis.

The prognostic value of the 29 deregulated miRNAs was also investigated in function of the overall survival and time to metastasis by subdividing the OS samples into high/low expression groups. This identified two miRNAs, miR‐221‐3p and miR‐222‐3p, in which the expression level showed a significant association with time to metastasis. When combining the high/low expression scores for the two miRNAs for each OS patient, the separation became even more distinct and significant, signifying the robustness of the miR‐221/222 signature as a prognostic biomarker. Increased expression of miR‐221 and miR‐222 has also been shown to be significantly associated with lymphatic metastasis in gastric, colorectal, and breast cancer (Falkenberg *et al*., 2013; Fu *et al*., 2014; Liu *et al*., 2012b; Sun *et al*., 2011). Furthermore, these two miRNAs have been shown to be a promising biomarker in breast cancer in function of time to developing distant metastasis, although a high expression of both miRNAs was associated with poor prognosis in breast cancer (Falkenberg *et al*., 2013). The discrepancy of miR‐222 expression between these studies and the OS samples in this study may be explained by the ability of a miRNA to target several hundred genes, thereby promoting tumor growth in some malignancies and repressing it in others. This is further substantiated by the fact that miR‐222 was shown to target *MMP1*, which is associated with increased aggressiveness and metastatic risk in oral tongue squamous cell carcinoma (Liu *et al*., 2009) (*MMP1* showed a 10‐fold increase in expression in the OS samples in this study). High expression of *MMP1* has also been associated with the development of metastasis in colorectal and breast cancer (Liu *et al*., 2012a; Sunami *et al*., 2000). miR‐221 targets among other genes *BCL2L11*, which exhibits pro‐apoptotic properties, thereby suppressing tumor growth (Aichberger *et al*., 2009), as well as the gatekeeper *PTEN* (Li *et al*., 2016). In different cancers, high expression of miR‐221/222 has also been shown to facilitate epithelial/mesenchymal transition, a prerequisite for metastasis formation (Li *et al*., 2015; Stinson *et al*., 2011). Furthermore, modulation of miR‐221 levels in OS cell lines demonstrated its role in carcinogenesis with high levels leading to increasing invasiveness and migration of the cells (Zhu *et al*., 2015). Hence, both the deregulation itself and the target genes of miR‐221 and miR‐222 indicate a prominent role of these two miRNAs in the ability of a neoplasm to spread to other organs and in the aggressiveness of specific cancer types.

Of the 29 deregulated miRNAs identified in this study, expression changes in nine miRNAs have not previously been associated with OS in miRNA profiling studies, while 20 miRNAs previously associated with OS development and progression were uncovered (Duan *et al*., 2011; Jones *et al*., 2012; Lulla *et al*., 2011; Maire *et al*., 2011; Namlos *et al*., 2012; Thayanithy *et al*., 2012; Won *et al*., 2013; Zhang *et al*., 2015). However, as OS is characterized by many different alterations, confirmation of previous findings is important for this particular disease. Furthermore, many of the formerly reported miRNA changes in OS have been identified in cohorts comprising less than 10 OS samples. Previous studies profiling miRNA expression changes in OS revealed 14 miRNAs significantly changed in these studies, which were not among the 29 miRNAs identified in our study. This may be due to differences in the cohorts, but also due to low sample number in the published studies or the use of normal bone as the control tissue, comprising a lower number than the OS cohorts (comprising 2–12 normal bone samples) (Jones *et al*., 2012; Namlos *et al*., 2012; Thayanithy *et al*., 2012; Won *et al*., 2013; Zhang *et al*., 2015). The cell of origin for OS has not yet been completely clarified. Both mesenchymal stem cells (MSCs) and derived osteoprogenitors such as OBs have been analyzed for their ability to form OS. Several studies have shown that OB precursors presented a higher incidence of OS compared to early mesenchymal progenitors (Abarrategi *et al*., 2016), supporting the model of OS originating from cells with OB commitment rather than immature MSCs. OB cell lines have therefore been used as the normal controls in this study, as well as in several previous studies (comprising 1–3 OB cell lines) (Duan *et al*., 2011; Lulla *et al*., 2011; Maire *et al*., 2011). Only one study has so far been using both normal bone tissue and OB cell lines as controls, but analyzed only 15 miRNAs previously identified to be differentially expressed between normal bone and OS cell lines. Nine of these were validated in primary OS samples when compared to normal bone. However, for some of these miRNAs, the authors observed opposite expression changes in OS when compared to either OB cell lines or normal bone (Namlos *et al*., 2012). This may be due to OS being at an intermediate state between the undifferentiated OB cells and the fully differentiated bones comprising several different cell types. Furthermore, normal bone was obtained from cancer patients suggesting a different age distribution between OS samples and bone samples, which could lead to differential miRNA expression profiles, and raise the question of the possible presence of cancer‐related epigenetic alterations in these samples. These points, as well as the small number of miRNAs analyzed in primary OS, bone and OB cell lines, make it difficult to draw general conclusions from this study. Even though OB cells can be considered to be the cell of origin of OS, the use of cell lines as normal control holds the limitation of identifying miRNAs with altered expression due to differences between cell lines and human tissue and not due to the cell origin. Unfortunately, no normal bone tissue was available for the current study. Therefore, future studies should comprise pediatric OB cells as normal control as well as normal bone to obtain a more comprehensive description of the role of the identified miRNAs in OS tumorigenesis.

Several of the 24 significantly downregulated miRNAs identified in this study have previously been identified as tumor suppressor miRNAs in other malignancies, among others miR‐100‐5p, miR‐125b‐5p, miR‐127‐3p, miR‐370‐3p, miR‐335‐5p, and miR‐411‐5p (Gao *et al*., 2016; He *et al*., 2013; Jiang *et al*., 2014; Li *et al*., 2014; Luan *et al*., 2016; Zhang *et al*., 2016). Of these, miR‐100‐5p, miR‐127‐3p, and miR‐335‐5p have previously been identified as downregulated in OS studies analyzing 2–18 samples (Jones *et al*., 2012; Maire *et al*., 2011; Thayanithy *et al*., 2012; Zhang *et al*., 2015). However, miR‐127‐3p and miR‐370‐3p have also been detected upregulated in two OS samples in one study (Zhang *et al*., 2015). miR‐125b‐5p and miR‐411‐5p have not previously been identified having an altered expression in any OS miRNA profiling studies. miR‐125b has been shown to be an important regulator of both proliferation and differentiation in different cell types (Scott *et al*., 2007; Sempere *et al*., 2004). Moreover, miR‐125b has been shown to inhibit normal OB proliferation in mouse cells (Mizuno *et al*., 2008), indicating a role of this miRNA in bone development and thereby in OS tumorigenesis. miR‐335‐5p has also been identified to be important in the differentiation process of bone cells, as a positive regulator of Wnt signaling through the inhibition of Wnt antagonists (Zhang *et al*., 2011). Wnt signaling is elevated when osteoprecursor cells differentiate into OBs. However, Wnt signaling is inhibited when the OB cells differentiate further toward the terminal stage of bone‐forming cells, which may be a functional explanation for the downregulation of miR‐335‐5p in OS.

Of the five significantly upregulated miRNAs identified in this study, miR‐181a‐5p, miR‐181c‐5p, miR‐223‐3p, and miR‐342‐3p have been described as oncomiRs in other cancer types (Mi *et al*., 2017; Tao *et al*., 2014; Walter *et al*., 2013; Wei *et al*., 2014). Of these, miR‐181a‐5p, miR‐181c‐5p, and miR‐223‐3p have previously been identified as upregulated in 4–18 OS samples (Jones *et al*., 2012; Lulla *et al*., 2011; Maire *et al*., 2011; Won *et al*., 2013). However, miR‐223‐3p has also been detected as downregulated in 18 OS samples in one study (Jones *et al*., 2012). miR‐342‐3p has not previously been associated with OS in miRNA profiling studies. miR‐181a‐5p has been shown to downregulate a Wnt antagonist, thereby activating the Wnt signaling, which is, as described above, important for the differentiation of osteoprecursor cells into OB cells (Lyu *et al*., 2017).

Future functional studies for each identified miRNA and their target genes in OS development and progression have a strong potential for discovering novel diagnostic and prognostic markers as well as developing new treatment strategies, thereby improving the long‐term survival rate for these young patients. Circulating miRNAs detected in serum and plasma of patients with cancer are being widely investigated for their potential as both diagnostic and prognostic markers of cancer, including OS (Ram Kumar *et al*., 2016). This might also provide a tool of diagnosing OS at an earlier time point when individuals complain repeatedly about ‘growing’ pains. Furthermore, miRNAs hold the promise of novel therapeutic targets, either by blocking the expression of an oncomiR or by substituting the loss of a tumor suppressor miRNA.

## 5 Conclusion

In conclusion, this study substantiates the importance of miRNA deregulation in OS and its association with OS tumorigenesis. We have identified and validated 29 deregulated miRNAs in, to our knowledge, the largest discovery and validation cohorts published, comprising a total of 101 OS samples. Both the miRNAs and their identified target genes are associated with multiple pathways and biological functions, related to cancer development and progression, as well as bone cell biology, thereby associating the deregulated miRNAs with OS tumorigenesis. Interestingly, a promising potential as prognostic biomarkers for the aggressiveness of OS was seen for two of these miRNAs (miR‐221‐3p and miR‐222‐3p).

## Author contributions

GBA collected the patient samples, performed experiments, analyzed the data, performed statistical tests, and wrote the manuscript. AK purified RNA from FFPE samples and made cDNA. HH provided patient information and confirmed the presence of tumor cells from HE‐stained sections. LLH provided supervision and assistance for writing the manuscript and designing figures and tables. JT provided supervision and assistance for data analysis and interpretation and for writing the manuscript. All authors read and approved the final manuscript.

## Supporting information


**Fig. S1.** Principal component analyses of the 339 detected miRNAs.

**Fig. S2.** Boxplots of the expression level of 24 differentially expressed miRNAs.

**Fig. S3.** Boxplots of the expression level of three miRNAs associated with the metastatic potential of OS; two miRNAs associated with the chemotherapeutic response of OS.

**Fig. S4.** Prognostic value of the expression of two miRNAs in function of time to metastasis.

**Table S1.** Raw expression data (C_q_ values) for 752 miRNAs analyzed with the microRNA Ready‐to‐Use PCR, Human panel I and panel II v2 (Exiqon, Denmark) using the OS discovery cohort.

**Table S2.** Log_2_ normalized data for the 752 miRNAs analyzed for the OS discovery cohort.

**Table S3.** Raw expression data (C_q_ values) for 33 miRNAs to be validated and the five reference miRNAs analyzed with the Pick&Mix microRNA PCR Panel, 384 well Ready‐to‐Use (Exiqon, Denmark) using the OS validation cohort.

**Table S4.** Log_2_ normalized data for the 33 miRNAs analyzed in the OS validation cohort.

**Table S5.** Expression of 301 upregulated and 60 downregulated genes identified to be targeted by the 29 deregulated miRNAs.

**Table S6.** Expression of 76 miRNAs identified as differentially expressed in OS samples when compared to OB cell lines.

**Table S7.** Previous miRNA profiling studies describing the 33 miRNAs identified in the discovery cohort.

**Table S8.** Comparison of profiling studies investigating miRNA expression changes in OS and their expression in the OS investigation cohort in this study.

**Table S9.** Expression of three miRNAs associated with the metastatic potential of OS and two miRNAs associated with the chemotherapeutic response of OS.
