# Supplementary figures and images for: miRNA profiling identifies deregulated miRNAs associated with osteosarcoma development and time to metastasis in two large cohorts
Source: Mol Oncol. 2017 Dec 1;12(1):114–31. doi: 10.1002/1878-0261.12154 (PMC5748490; doi:10.1002/1878-0261.12154)

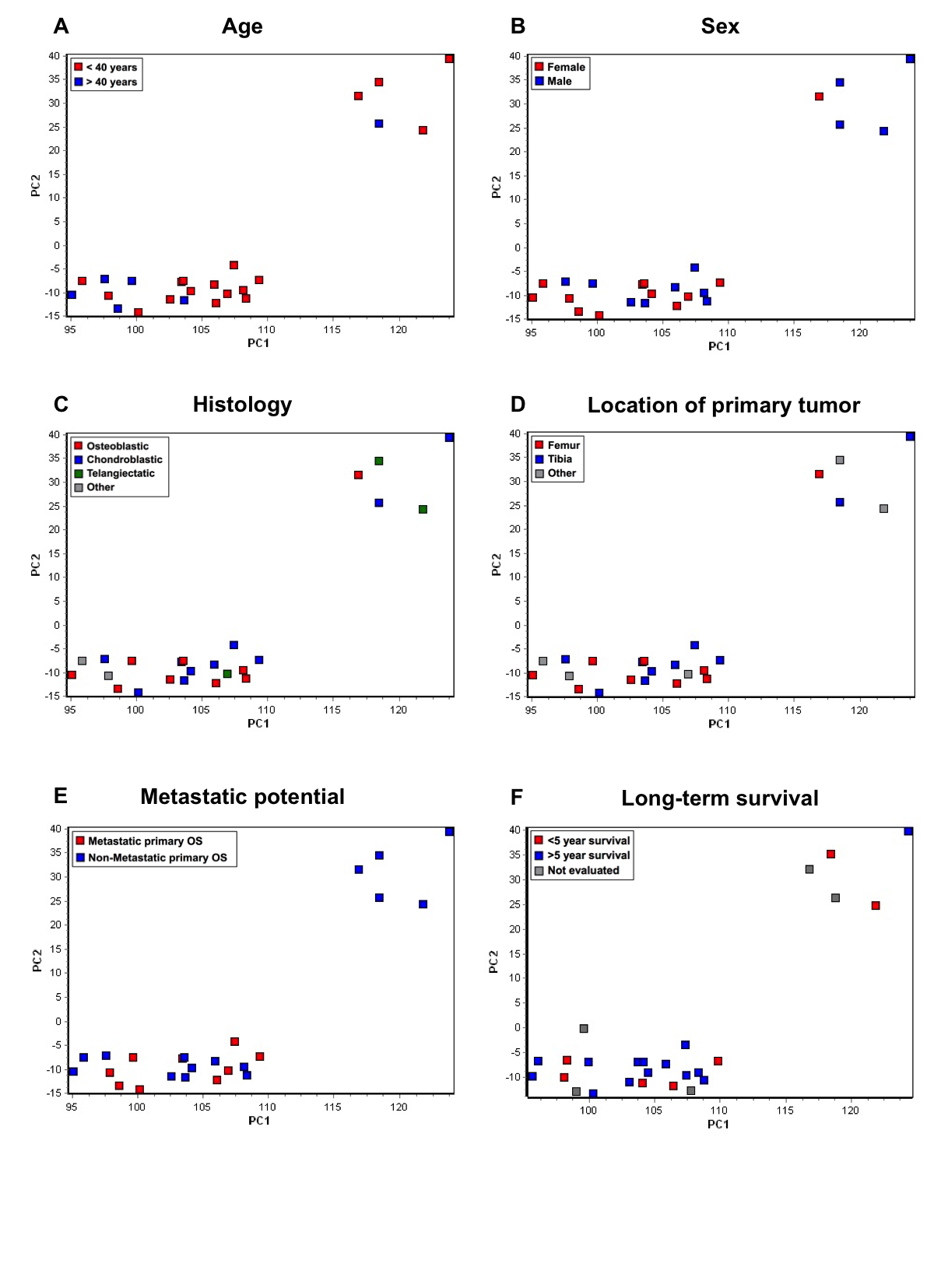

Supplement: Supplementary file 1 — Fig. S1. Principal component analyses of the 339 detected miRNAs. [file MOL2-12-114-s001.tiff]
